# Aging increases flexibility of postural reactive responses based on constraints imposed by a manual task

**DOI:** 10.3389/fnagi.2014.00327

**Published:** 2014-12-03

**Authors:** Andrea Cristina de Lima-Pardini, Daniel Boari Coelho, Marina Brito Silva, Nametala Maia Azzi, Alessandra Rezende Martinelli, Fay Bahling Horak, Luis Augusto Teixeira

**Affiliations:** ^1^Laboratory of Medical Investigation (LIM44), Institute of Radiology, Faculty of Medicine, University of São PauloSão Paulo, SP, Brazil; ^2^Human Motor Systems Laboratory, Department of Human Movement Biodynamics, School of Physical Education and Sport, University of São PauloSão Paulo, SP, Brazil; ^3^Portland VA Medical Center and the Balance Disorders Laboratory, Department of Neurology, School of Medicine, Oregon Health and Science UniversityPortland, OR, USA

**Keywords:** postural control, manual constraint, dual task, elderly, postural adaptation, balance control

## Abstract

This study compared the effect of stability constraints imposed by a manual task on the adaptation of postural responses between 16 healthy elderly (mean age = 71.56 years, SD = 7.38) and 16 healthy young (mean age = 22.94 years, SD = 4.82) individuals. Postural stability was perturbed through unexpected release of a load attached to the participant’s trunk while performing two versions of a voluntary task: holding a tray with a cylinder placed with its flat side down (low constraint) or with its rolling round side down (high constraint). Low and high constraint tasks were performed in alternate blocks of trials. Results showed that young participants adapted muscular activation and kinematics of postural responses in association with previous experience with the first block of manual task constraint, whereas the elderly modulated postural responses based on the current manual constraint. This study provides evidence for flexibility of postural strategies in the elderly to deal with constraints imposed by a manual task.

## Introduction

Given the emotional, physical, and economical burden of falls in the elderly, falls are one of the most concerning problems of the geriatric field. Recent studies showed that the prevalence of falls in people aged >65 years is over 30%, reaching more than 50% in people aged >80 years (Costello and Edelstein, 2008; de Negreiros Cabral et al., 2013). One of the most critical situations that could lead to a fall in an elderly individual is the accomplishment of a manual task during the requirement of body balance. For example, holding a glass of water significantly decreases the speed of gait and could be an useful predictor of future falls in the elderly (Lundin-Olsson et al., 1998). The difficulty of coupling a manual task with the postural control task demonstrates in this population an impaired interaction of automatic processing of upright balance with the constraints of the context, which is processed by high order centers of the central nervous system (CNS).

Fast postural reactive responses to an unpredictable perturbation have been a useful tool to assess the ability to adapt postural control to different contexts. Although occurring in less than one fifth of a second, postural reactive responses are shown to be influenced by the context, involving high-order process in the brain (Horak et al., 1989a; Chong et al., 2000; Taube et al., 2006; Jacobs and Horak, 2007; Jacobs et al., 2008; de Lima et al., 2010). The influence of high order processing on automatic postural control is important for the flexibility of postural reactive responses to specific contexts. For example, recent studies have shown different manifestations of postural reactive responses when the constraints of a manual task were manipulated in humans (Oullier et al., 2004; de Lima-Pardini et al., 2012; Papegaaij et al., 2012). Studies suggest that manual task constraints are associated with the neural effort involved to perform the task (Johansen-Berg and Matthews, 2002), influencing postural control (Bardy et al., 1999; Oullier et al., 2004; de Lima et al., 2010). These results are in accordance with the idea that different levels of movement control (automatic and voluntary) interact to adapt postural reactive responses to the context, which is known as “postural set” (Prochazka, 1989). Some studies showed that elderly compared to young people do not totally suppress activation of the gastrocnemius medialis (GM) muscle when the tibialis anterior (TA) is activated, after sudden switching of the direction of the base of support (Woollacott et al., 1986; Chong et al., 2000). Furthermore, the elderly activate unnecessary muscles when using an ankle strategy and show less modulation of muscle responses to a slippery surface (Horak et al., 1992). Such findings suggest that aging decreases the ability to modify postural reactive responses based on the requirements of specific contexts, implying less flexibility of postural set.

Despite conclusions that the elderly have impairments in postural set (Woollacott et al., 1986; Horak et al., 1992; Chong et al., 2000), there is a paucity of studies specifically addressing the adaptation of postural control to the constraints of a manual task in this population. Neurophysiological and biomechanical analyses on the interaction of two distinct levels of processing (automatic and voluntary) related to postural and manual control in dynamic situations should be investigated in greater detail in the elderly. To the best of our knowledge, only our previous two studies have addressed the interaction between posture and the constraint level of a manual task in elderly people during perturbations of body equilibrium. de Lima-Pardini et al. (2012) and Papegaaij et al. (2012) investigated the influence of manual constraint on postural reactive responses during unpredictable, backward translations of the support surface in the elderly. The elderly subjects were to hold a tray with a cylinder on it with the flat side down (low constraint) or with a round side down (high constraint). The main results showed that muscular responses, margin of stability and joint coordination were modulated according to the constraint of the manual task. However, in these studies, responses in elderly individuals were not compared to a young group, which hampers the interpretation of the integrity of manual and postural interactions with aging.

In the present study, we hypothesize that, compared to young individuals, the elderly will show less flexibility of postural reactive responses to different levels of manual task constraint, implying impairment in the interaction between higher and lower levels of CNS processing in the elderly. Besides kinetic and electromyography (EMG) of some postural muscles, we addressed the analysis of joint coordination among shoulder, knee and ankle that could elucidate in more detail the influence of manual task constraint on body posture.

## Methods

### Participants

Sixteen healthy physically active elderly (8 females), age range from 61 to 82 years (*M* = 71.56, SD = 7.38), and 16 young adults (8 females), age range from 17 to 32 years (*M* = 22.94, SD = 4.82), participated in this study. Participants were screened for physical and neurological dysfunctions that might impair postural control. Elderly participants were assessed with the Mini Mental State Examination (Folstein et al., 1975) for evaluation of cognitive function. All participants provided informed consent, and the local university Ethics Committee approved experimental procedures.

### Apparatus and task

**Figure 1:**
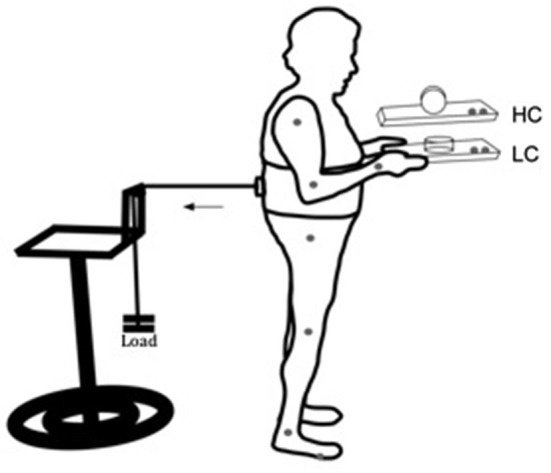
**Schematic representation of the experimental setup for the low (LC) and high (HC) constraint conditions**. Gray dots at the joints and at the tray represent kinematic markers. The horizontal arrow indicates direction of the traction exerted by the load pulling the participant’s trunk backward.

Participants performed a dual-task, combining a perturbed upright stance with a manual task involving voluntarily balancing a movable cylinder (diameter 9 cm, height 5 cm, 100 g) on a wooden tray (24 × 33 cm, 350 g). The manual component of the task consisted of keeping the upper arms parallel to the trunk and elbows bent at approximately 90°, while the hands were supinated to hold the tray (Figure 1). The aim of the manual task component was to keep the cylinder on the tray as motionless as possible in response to postural perturbation. In the low constraint condition (LC), the cylinder was lying on its flat side, so that slow to medium horizontal movements of the tray would not lead to sliding of the cylinder on the tray (Figure 1, LC). In the high constraint condition (HC), the cylinder was placed on its round surface, so that it was free to roll in the anterior-posterior direction, limited in motion to around 90° by a weight of 10 g attached to the internal side of the cylinder (Figure 1, HC).

Participants wore a harness positioned at the lumbar-sacral region. On the posterior side of the harness an electromagnetic system was coupled to a load through a steel cable, passing through a pulley attached to a height adjustable support. With that arrangement the pulling force applied to the subject’s torso through the apparatus was approximately horizontal with the ground. The load had approximately 7% of the subjects’ weight, and it was used to pull the participant’s trunk backward. The posterior pulling force applied through the load required a constant activation of anterior muscles. Load was unexpectedly released by the experimenter through a remote switch. Load release induced a forward postural sway, requiring the establishment of a new point of postural equilibrium through a reactive phasic contraction of the posterior muscles of the body. The aim of the postural component of the task was to maintain a stable upright stance, before and after load release. Ground reaction forces were recorded through a force plate (AMTI, OR6-WP) on which the subjects stood.

### Experimental design

Both young and elderly groups were evaluated in two experimental conditions, low and high constraint. The experimental protocol consisted of three blocks of seven trials under each task constraint, for a total of 42 trials. Low and high constraint trials were alternated between blocks, with sequence of tasks counterbalanced across participants within groups. This blocked design allowed the assessment of the sequence effect. Repetition of trials under the same task constraint within blocks allowed for assessment of inter-trial adaptability, while alternation between different block conditions allowed for assessment of between-task adaptability.

### Procedures

An assistant stood near the participant to assist in cases of loss of balance. To avoid an anticipatory forward tilt of the body to compensate for the force pulling the trunk, a plumb line was positioned at the right side of the participant. To assume appropriate postural position, the plumb line passed approximately through the center of the ear canal, shoulder, greater trochanter, knee and slightly in front of the lateral malleolus. Before each trial, the experimenter verified whether the participant was positioned appropriately in relation to the plumb line. Participants were informed at the beginning of each trial that the load release could occur anytime within a window of 10 s. The following instructions were given to participants: “stand straight with your upper arms parallel to your body, keeping your elbows at 90° and gazing at the cylinder. The load will be released. We will not tell you to get ready, so you will not know exactly when the perturbation will be applied. Please, keep gazing at the cylinder and try to avoid any movement of the cylinder. Also, try not to step during the perturbation.” Participants were asked to place their feet in a comfortable position on the force platform. Feet positions were marked with tape to make sure the same position was maintained throughout the experiment. No familiarization trials before the experiment were provided. On finishing each trial, participants returned the tray to the experimenter and waited until the next trial. Inter-trial intervals were about 20 s and rest intervals lasting 1 min were provided between blocks. After three blocks, a longer rest of 15 min was allowed, interval during which participants stayed sat. Trials in which participants stepped out the platform or talked were canceled out and were not repeated (1.64% of elderly trials and 0.15% of younger trials were excluded). Feedback was not provided to the participants either during or between the trials.

### Data collection and analysis

Muscular activation was measured via EMG from the medial head of right GM and TA, using 2.5 cm self-adhesive gel-filled bipolar Ag–AgCl surface electrodes attached 2–4 cm apart. The skin was shaved, cleaned and scrubbed prior to application of the electrodes. Online, EMG signals were amplified at a gain of 1000, band-pass filtered from 20–400 Hz, and sampled at 1000 Hz. Offline, they were rectified and low-pass filtered at 10 Hz. Center of pressure (CoP) position over time was sampled from the force plate with a frequency of 150 Hz and low-pass filtered at 10 Hz. Angular displacement of the ankle, hip and shoulder were measured with passive markers attached at the following anatomical points on the right body side: fifth metatarsophalangeal joint, heel, lateral malleolus, lateral knee joint center, greater trochanter, acromion, lateral epicondyle, and wrist joint center. Two markers were also attached on the tray to track its horizontal displacement (Figure 1). Marker positions were tracked using a six-camera optoelectronic motion analysis system (VICON, MX3+). Kinematic data were sampled at 200 Hz.

Dependent variables were the following:

#### Maximum tray velocity

This variable was used to compare performance of the voluntary task between the LC and HC conditions. This was calculated as the maximum linear velocity in the ensuing 500-ms interval after postural perturbation (load release). Low tray velocity was interpreted as better performance in the voluntary task.

#### Anterior displacement of the center of pressure (CoP)—postural sway

This variable was used to compare performance of the postural task between the LC and HC conditions. Postural sway was quantified as the difference between the maximum anterior displacement (anterior peak) after the postural perturbation and the mean of anterior-posterior position 200 ms before load release (baseline). The length of the right foot of each participant was used to normalize CoP.

Postural reactive responses were also quantified as magnitude of activation of the GM and TA muscles, and their co-activation. Muscle burst onset was identified as the first sustained (>25 ms) EMG activity greater than two standard deviations above the baseline. Magnitude of initial postural response was defined as the integrated EMG in the time interval of 75 ms after GM activation onset (Horak et al., 1996; Papegaaij et al., 2012) for both muscles. Electromyography values were normalized by its maximum value in the period between 100 ms before and 500 ms after the perturbation for each trial. The coactivation index was based on the normalized magnitude of the EMG signals according to the following equation (Ervilha et al., 2012):

(2*TA/TA+GM)*100

Interaction between the voluntary and postural tasks was characterized by the coordination among the shoulder, hip and ankle joints (de Lima-Pardini et al., 2012). Interjoint coordination was assessed by fitting an ellipse embracing 95% of the values of the angle-angle plot for joint displacement, from the perturbation onset until the end of the trial. A regression analysis was conducted to find the slopes of the fitted ellipses representing the coordination between shoulder and hip and between hip and ankle. For the slopes representing the coordination between shoulder (x-axis) and hip (y-axis), higher slopes indicate relatively larger participation of the hip, and lower values indicate relatively larger participation of the shoulder. For the slopes representing the coordination between hip (x-axis) and ankle (y-axis), higher slopes indicate relatively larger participation of the ankle, and lower values indicate relatively larger participation of the hip. Relative phases between shoulder and hip joints and between hip and ankle joints were calculated with the cross-spectral density method (Bennett et al., 2005). The values of relative phase ranged from 0 to 180°, which can show the kind of the coordination among the joints—in phase (near 0°), anti-phase (near 180°) or out-phase (other phase relations between joints motion). Considering the balance requirement of the manual task, an out-phase relation between shoulder and hip and between hip and ankle joints is expected.

### Statistical analysis

Parametric requirements for normality were verified through the Kolmogorov-Smirnov test. Adaptation across blocks was measured for all variables, comparing the averages obtained in the first block with the last block of trials. Data were preliminarily analyzed through a four-way 2 (group) × 2 (sequence: low-high [L-H] × high-low [H-L]) × 2 (context: HC × LC) × 2 (block: first × last) ANOVAs with repeated measures on the last two factors. Results showed absence of block effect for all variables. For this reason, three-way 2 (group) × 2 (sequence) × 2 (constraint) ANOVAs with repeated measures on the constraint factor were used. The level of significance was set at 0.05. *Post hoc* comparisons were made using the Fisher test.

## Results

**Figure 2:**
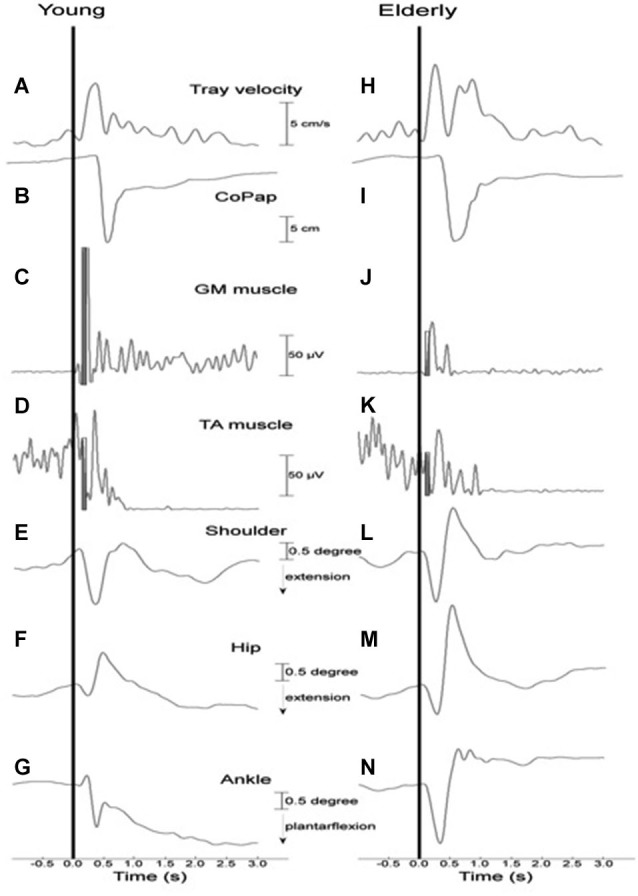
**Representative curves of a young (A–G) and an elderly (H–N) participant in a high constraint current context and sequence for tray velocity (A,H), center of pressure displacement (B,I), activation of gastrocnemius medialis (C,J) and tibialis anterior (D,K) muscles, angular displacement of shoulder (E,L), hip (F,M) and ankle (G,N) joints**. Vertical line indicates time of load release. The filled rectangles **(C,D,J,K)** represent the time interval of 75 ms after the onset of the GM muscle that was used to calculate the muscular magnitude.

Figure 2 depicts tray velocity, CoP sway, EMG for the GM and TA muscles, and shoulder, hip, and ankle joint angular displacements of one representative subject for each experimental group in the HC context.

### Tray velocity

**Figure 3:**
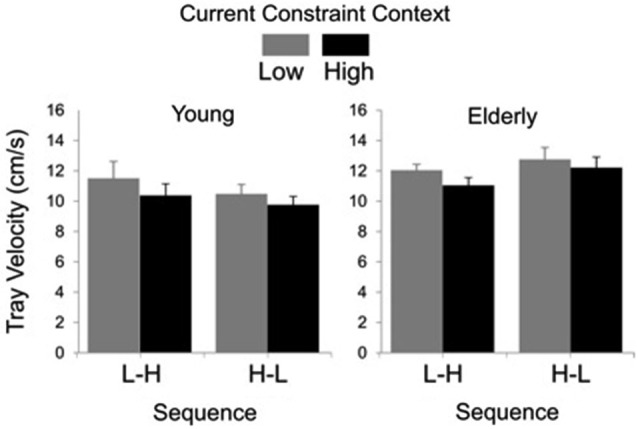
**Tray velocity averages (standard errors represented by vertical bars) as a function of current constraint condition and constraint sequence for the young and elderly groups**.

In Figure 3, groups’ averages for tray velocity are compared as a function of task constraint and sequence. Analysis showed significant main effects of group (*F*_(1, 27)_ = 4.69, *P* < 0.01) and constraint (*F*_(1, 27)_ = 20.94, *P* < 0.01). The main effect of group was due to lower tray velocity in the young (*M* = 10.47 cm/s, SE = 0.40) than for the elderly group (*M* = 11.98 cm/s, SE = 0.31). The main effect of constraint was due to lower tray velocity in the high (*M* = 10.84 cm/s, SE = 0.35) than in the low constraint (*M* = 11.67 cm/s, SE = 0.40).

### Postural reactive response

Analysis of CoP did not show any significant main effect of group (*F*_(1,28)_ = 0.72, *P* = 0.40) (young: *M* = 12.03 cm, SE = 0.42; elderly: *M* = 11.26 cm, SE = 0.42), sequence (*F*_(1,28)_ = 0.82, *P* = 0.37) (L-H: *M* = 11.77 cm, SE = 0.40; H-L: *M* = 11.23, SE = 0.43) or constraint (*F*_(1,28)_ = 3.30, *P* = 0.08) (LC: *M* = 11.38 cm, SE = 0.42; HC: *M* = 12.96 cm, SE = 0.43).

**Figure 4:**
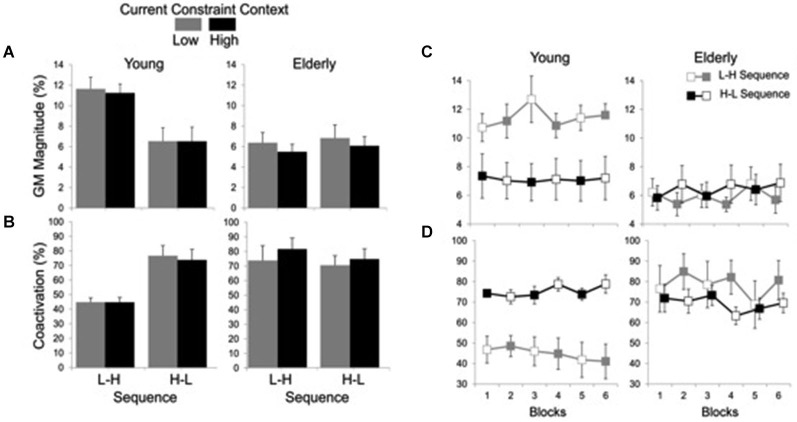
**Averages (standard errors in vertical bars) of GM activation magnitude (A) and coactivation between GM and TA muscles (B) for current constraint and constraint sequence for the young and elderly groups**. Panel **(C)** represents GM magnitude and panel **(D)** shows GM-TA coactivation across blocks of trials, representing high constraint by filled squares and low constraint by open squares. Note the effect of sequence constraint in the young group vs. significant effect of current constraint in the elderly group.

Averages and time series of the magnitude of GM muscle activation and coactivation between GM and TA muscles are shown in Figure 4. Analysis of the magnitude of GM activation indicated significant main effects of group (*F*_(1, 19)_ = 6.94, *P* < 0.05) and constraint (*F*_(1, 19)_ = 4.94, *P* < 0.05), and a group by sequence interaction (*F*_(1, 19)_ = 6.60, *P* < 0.05). The effect of group was due to smaller GM activation for the elderly (*M* = 6.14%, SE = 0.46) than for the young (*M* = 9.36%, 0.73) group, while the effect of constraint was due to smaller GM activation in HC (7.57%, 0.67) than LC (8.10%, SE = 0.72) conditions. *Post hoc* comparisons for the group by sequence interaction showed that the H-L sequence resulted in smaller GM activation (*M* = 6.50%, SE = 0.89) than the L-H (*M* = 11.41%, SE = 0.69) sequence, but only for the young subjects.

Figure 4A suggests that while the young group’s postural responses were primarily affected by sequence, the elderly group’s postural responses were primarily  affected  by constraint. For  this  reason,  each  group  was  submitted  separately  to   an   additional two-way 2 (sequence) × 2 (constraint) ANOVA with repeated measures on the second factor. Results for the elderly group showed a significant main effect of constraint (*F*_(1,9)_ = 9.50, *P* < 0.05) due to smaller GM activation in the HC (*M* = 5.73%, SE = 0.55) than in the LC (*M* = 6.58%, SE = 0.75) conditions. Results for the young group, on the other hand, showed a significant main effect of sequence (*F*_(1,10)_ = 9.44, *P* < 0.05), with smaller GM activation magnitude in H-L sequence (*M* = 6.50%, SE = 1.26) than in the L-H sequence (*M* = 11.41%, SE = 0.97).

Coactivation analysis indicated significant main effects of group (*F*_(1, 20)_ = 5.42, *P* < 0.05) and constraint (*F*_(1, 20)_ = 4.46, *P* < 0.05), and group by sequence (*F*_(1, 20)_ = 6.74, *P* < 0.05) and group by constraint (*F*_(1, 20)_ = 11.83, *P* < 0.01) interactions. The main effect of group was due to greater muscle coactivation in the elderly (*M* = 76.03%, SE = 4.03) as compared to the young (*M* = 57.53%, SE = 3.87). The coactivation was also greater in HC (*M* = 68.01%, SE = 4.40) than in LC (*M* = 65.55%, SE = 4.40) conditions. *Post hoc* comparisons for the group by sequence interaction showed that for the young the H-L (*M* = 75.30%, SE = 4.76) led to greater coactivation than the L-H (*M* = 44.84%, SE = 2.13) sequence, while no significant difference in muscle coactivation between sequences was found for the elderly group (L-H: *M* = 78.60%, SE = 6.71; H-L: *M* = 73.46%, SE = 4.67). *Post hoc* comparisons for the group by constraint interaction showed that the high constraint led to greater coactivation (*M* = 79.11%, SE = 5.30) than the low constraint (*M* = 72.95%, SE = 6.18) conditions for the elderly group, while no significant difference in muscle coactivation between constraints was found for the young group (LC: *M* = 58.15%, SE = 5.70; HC: *M* = 56.91%, SE = 5.50) (Figure 4B).

### Shoulder-hip and hip-ankle coordination

**Figure 5:**
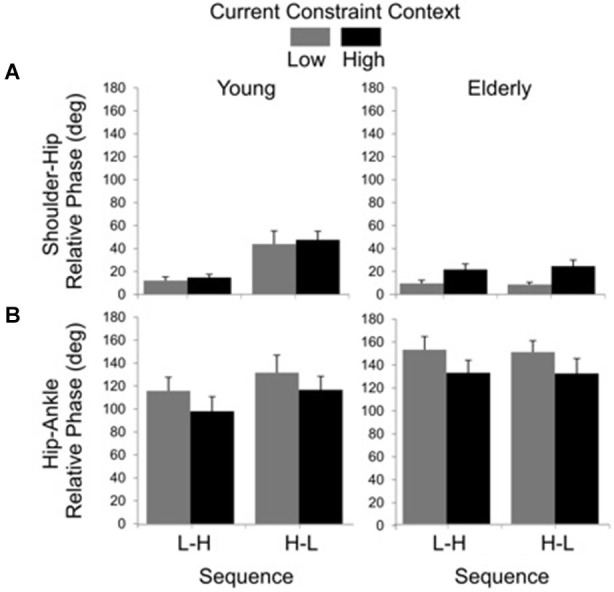
**Averages (standard errors in vertical bars) for shoulder-hip (A) and hip-ankle (B) relative phases as a function of current constraint and constraint sequence for the young and elderly groups**.

Results for relative phase between shoulder and hip angular displacements showed significant main effects of group (*F*_(1, 19)_ = 7.98, *P* < 0.01), sequence (*F*_(1, 19)_ = 12.24, *P* < 0.01) and constraint (*F*_(1, 19)_ = 11.75, *P* < 0.01), as well as significant group by sequence (*F*_(1, 19)_ = 10.87, *P* < 0.01) and group by constraint (*F*_(1, 19)_ = 4.68, *P* < 0.05) interactions. The main effect of group was due to larger values of relative phase in the young (*M* = 27.70 deg, SE = 4.678) than in the elderly (*M* = 15.74 deg, SE = 2.39) group. The sequence effect was due to smaller values of relative phase in the L-H (*M* = 14.14 deg, SE = 1.90) than in the H-L (*M* = 29.45 deg, SE = 4.70) sequence. The constraint effect was due to larger relative phase in the HC (*M* = 25.86 deg, SE = 2.47) than in the LC (*M* = 17.07 deg, SE = 2.77) condition. *Post hoc* comparisons for the group by sequence interaction showed that the H-L led to larger relative phase (*M* = 45.32 deg, SE = 6.49) than the L-H sequence (*M* = 13.02 deg, SE = 2.10) for the young, but not for the elderly (L-H: *M* = 15.26 deg, SE = 3.24; H-L: *M* = 16.22 deg, SE = 3.65). *Post hoc* comparisons for the group by constraint interaction showed that high constraint led to larger relative phase (*M* = 22.74 deg, SE = 3.53) between shoulder and hip displacements than the low constraint (*M* = 8.74 deg, SE = 1.60) for the elderly, but not for the young (LC: *M* = 26.14 deg, SE = 7.20; HC: *M* = 29.26 deg, SE = 6.25; Figure 5A).

The relative phase between hip and ankle angular displacement showed significant main effects of group (*F*_(1, 24)_ = 4.48, *P* < 0.05) and constraint (*F*_(1, 24)_ = 10.78, *P* < 0.01). The effect of group was due to larger relative phase values in the elderly (*M* = 140.87 deg, SE = 6.25) than in the young (*M* = 114.96 deg, SE = 6.69). The effect of constraint indicated that the low constraint induced larger relative phases (*M* = 135.88 deg, SE = 6.81) than the high constraint (*M* = 118.10 deg, SE = 6.72) conditions (Figure 5B).

**Figure 6:**
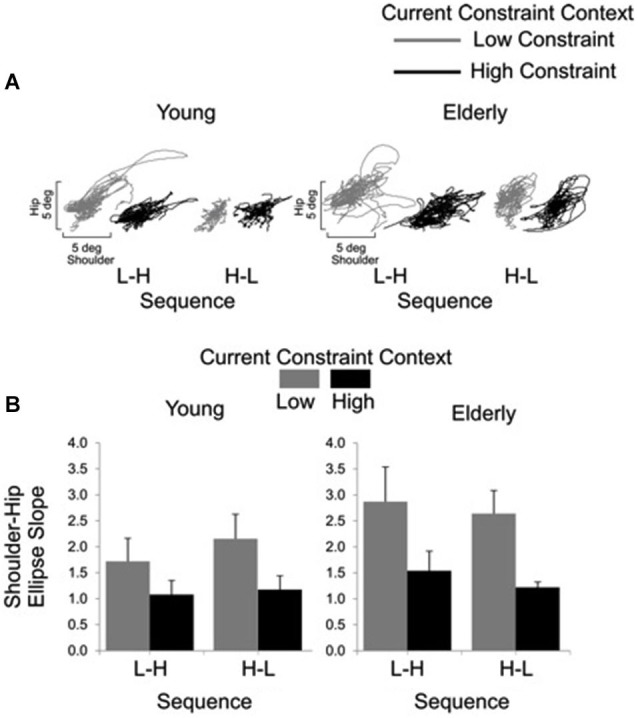
**Interjoint coordination between shoulder and hip for young and elderly participants as a function of current constraint and sequence constraint**. Representative examples of shoulder and hip coordination of 21 trials for young and for elderly subjects **(A)**. Averages (standard errors in vertical bars) for shoulder-hip ellipse slope as a function of current constraint and constraint sequence for young and elderly groups **(B)**.

**Figure 7:**
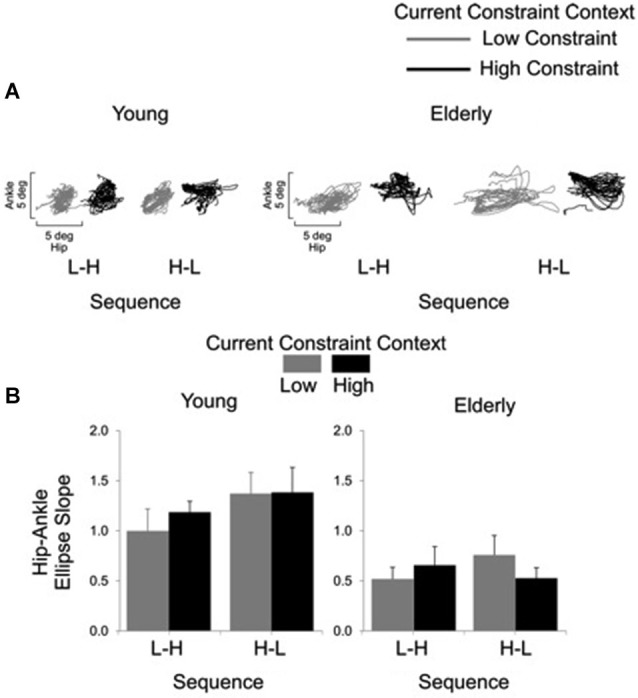
**Interjoint coordination between the hip and ankle joints for the young and the elderly as a function of current constraint and sequence constraint**. Representative examples of hip and ankle coordination of 21 trials for young and elderly subjects **(A)**. Averages (standard errors in vertical bars) for the hip-ankle ellipse slope as a function of current constraint and constraint sequence for young and elderly groups **(B)**.

Representative examples of shoulder-hip and hip-ankle joint angle plots are shown in Figures 6, 7, respectively. Analysis of ellipse slopes for the shoulder-hip joint angle indicated a significant main effect of constraint (*F*_(1, 27)_ = 31.85, *P* < 0.01). That effect was due to smaller values in the HC (*M* = 1.25 deg, SE = 0.13) than in the LC (*M* = 2.36 deg, SE = 0.26) condition (Figure 6B). Analysis of ellipse slopes for the hip-ankle joint angle indicated a significant main effect of group. That effect was due to smaller values in the elderly (*M* = 0.61 deg, SE = 0.08) than in the young (*M* = 1.23 deg, SE = 0.11; Figure 7B).

## Discussion

The primary result of this study refuted our hypothesis that the elderly have less flexible postural reactive responses due to the manual task constraint than the young. In fact, the elderly showed more consistent immediate adaptation of postural reactive responses to the current context of the manual constraint, whereas the young subjects were more affected by the prior context (i.e., relative sequence) of manual constraint.

Both groups adapted tray velocity to the current constraint imposed by the voluntary motor task. This result agrees with our prior studies with elderly subjects (de Lima et al., 2010; de Lima-Pardini et al., 2012; Papegaaij et al., 2012). However, unlike our earlier studies, the present study compared the performance of the elderly with a young group, to determine the aging effect. Adapting the manual task to its constraints demonstrates that the elderly are able to adopt different voluntary strategies, as the young do, specifically during dynamic postural conditions. Conversely, in previous studies investigating manual performance during quiet stance, the elderly showed poorer grip-load coordination, compared to their young counterparts (Augurelle et al., 2002; Gorniak and Alberts, 2013). However, to compensate for the deterioration of grip-load coordination, the elderly in these studies decreased movement time, which was possible due to their lifetime of practice in performing manual tasks (Gilles and Wing, 2003; Danion et al., 2007; Gorniak and Alberts, 2013). Similarly, in the present study elderly subjects were able to successfully perform a manual task requiring a high level of skill by increasing tray velocity (decreasing of movement time) as a compensatory strategy, and this change in movement time was larger than in the young group. Also, the analysis of the coordination between shoulder and hip (ellipse slope) indicated that for both groups the HC current condition led to larger displacement of the shoulder relative to the hip. Larger displacement of the shoulder could be attributed to the requirement of higher stability demands of the HC manual task. Interestingly, the results also showed that hip-ankle coordination (relative phase) was modulated in both age groups based on the current task constraint evidencing the coupling between the requirements of the manual task and the adjustments of the body.

The elderly subjects primarily adopted the hip strategy (smaller hip-ankle slope) in response to postural perturbations, as reported previously (Horak et al., 1989b). Studies have demonstrated that the hip strategy is optimal for quickly moving the center of mass (Horak et al., 1997). However, in this case, there is an associated trunk flexion that could negatively affect maintenance of stability for the manual task. Under this situation of increased trunk flexion, the elderly may have adopted the greater tray velocities as a compensatory strategy to maintain performance of the voluntary manual task. Considering that the requirements of manual stability assessed in our study are strictly dependent on the maintenance of postural stability, one could infer that the adoption of compensatory manual strategies by the elderly involved the interaction between upper limb control with compensatory postural reactive responses.

Both groups maintained similar balance sway, regardless of the constraint condition, consistent with our previous studies (de Lima-Pardini et al., 2012; Papegaaij et al., 2012). No participant demonstrated a high level of postural instability or lost balance as a result of perturbations during quiet stance. However, we did not see reduced postural sway during performance of a high constraint manual task as shown previously in young subjects during unperturbed stance. These studies support the notion that the stability demands from the voluntary task can lead to postural sway modulation in order to achieve better voluntary task performance (Stoffregen et al., 2000; Haddad et al., 2013). Muscle activation revealed a different type of adaptation strategy for each age group. The young subjects modulated their postural muscle responses based on prior experience with the manual constraint. The first trials of each condition defined the behavior of the subsequent trials, i.e., motor strategies were not shifted according to changes in the current constraint. On the other hand, in the elderly subjects’ magnitude of postural muscle activation and intermuscular coactivation were more sensitive to the current constraint conditions.

The elderly subjects reached a smaller shoulder/hip relative phase than the young. This finding could be interpreted as a difficulty of the elderly to uncouple shoulder and hip as compared to their younger counterparts. Under challenging conditions of postural control and simultaneously manual task performance, uncoupling the shoulder and hip joints would be an advantageous strategy to accomplish the task, considering that keeping the tray at the same spatial location during base of support translation would decrease its displacement. Thus, the age groups differed in postural adaptation strategies to respond to balance perturbation in association with the manual task constraint.

The elderly showed more specific postural adaptation to the current level of manual constraint than the young subjects. In contrast, the young subjects adopted a more general adaptation based on the first constraint condition. The type of adaptation (general or specific) of postural responses has been previously studied in young and elderly individuals who were submitted to continuous movements of the support basis, with serial and randomized perturbations (Van Ooteghem et al., 2008, 2009, 2010). In these studies, however, both young and elderly people were shown to adopt a general, rather than specific, adaptation to the postural perturbations. The authors concluded that less adaptation specificity is advantageous to postural control, inasmuch as it may represent lower risk in the case of transfer to a new context. Van Ooteghem et al. (2008) proposed that the specificity of the postural response could overload neural processing, impairing its ability to respond efficiently to unexpected postural perturbations. In this case, motor responses tend to adopt intermediate default values in similar conditions of perturbation (Horak et al., 1989a; Beckley et al., 1991; Van Ooteghem et al., 2008, 2009, 2010). A main conclusion by Van Ooteghem et al. (2010) is that aging does not affect generalized postural adaptation to unexpected postural perturbation. Nonetheless, our results support the opposite idea that aging leads to less generalized postural adaptation. The contradictory conclusions could be due mainly to the addition of manual task constraints. Accomplishment of a manual task predominantly influences the margin of postural stability (Riccio and Stoffregen, 1988; Riccio, 1992; Haddad et al., 2013). In this case, postural control is more constrained, involving alternative motor strategies for interaction between voluntary and postural control. Prior investigations have indicated that postural impairments do not manifest unless a challenging posture is adopted (Riccio, 1992; Nachreiner et al., 2007; Haddad et al., 2013). Considering that generalization of postural responses is a general representation of the environment used in planning the postural responses (feedforward control), being nonspecific to the current sensory state, under challenging postural conditions, the elderly subjects are less able to adopt a feedforward control of postural reactive responses.

We found that the elderly had smaller GM activation and greater intermuscular coactivation than the young. This finding could be related to decreased efficiency of lower circuits with aging, different brain recruitment patterns, recruitment of additional brain areas (Seidler et al., 2010; Fujiwara et al., 2012), greater participation of supraspinal structures and decrease in presynaptic inhibition by corticospinal circuits on postural muscles (Seidler et al., 2010; Papegaaij et al., 2014). In fact, balance maintenance in the elderly has been found to be more attentionally-demanding, i.e., less automatized, compared to young individuals (Cordo and Nashner, 1982; Horak et al., 1984; Brown et al., 1999; Brauer et al., 2001; Woollacott and Shumway-Cook, 2002). Increased participation of attention in postural control in the elderly could explain why they did not show generalization of postural responses as the young did, adapting to the current manual constraint.

Our experimental task required a balance of neural control between the automatic control that affords efficiency of postural responses to unexpected perturbations and the control of the voluntary task, which provides efficacy (responses according to the demands of the environment). It may be possible that the generalized postural responses shown by the young are a manifestation of the intact balance between efficiency and efficacy of their postural responses, considering that they were influenced by the manual task, but did not change responses at each changing of condition, thus, avoiding errors (Van Ooteghem et al., 2008). Following this idea, the increased influence of supraspinal circuits on postural control in the elderly might have impaired the balance of automaticity and context, feedforward influence, resulting in a greater weight of the context on postural responses that was evidenced by the effect of current constraint on muscular activation. Although apparently less efficient, elderly’s postural reactive response are more flexible to the constraints of the manual task than postural responses by the young.

Worth noting is the similarity of muscular activation between the young in the H-L sequence with the elderly in all conditions. This result could be related to the fact that increased challenging of a manual task decreases the boundaries of postural stability in young adults (Riccio, 1992; Haddad et al., 2013), affecting their muscular responses, simulating an aging effect (Riccio, 1992; Haddad et al., 2013). Prior studies have already shown that smaller postural muscular activation and higher coactivation are characteristics of postural responses of the elderly (Manchester et al., 1989; Woollacott and Shumway-Cook, 1990; Lin and Woollacott, 2005; Halická et al., 2012). Greater intermuscular coactivation has been shown to be a feature of aging, stiffening the ankle joint, and limiting the degrees of freedom during high postural demands (Manchester et al., 1989; Woollacott and Shumway-Cook, 1990; Halická et al., 2012). Smaller muscular activation in the elderly than the young subjects is thought to be a result of loss of muscle spindles, degeneration of afferent and efferent pathways and structural changes in cortical neuron (Papegaaij et al., 2014). We propose that, under a high constraint condition, postural control in the young becomes similar to postural control in the elderly.

## Conclusions

The results of the present study indicate that aging leads to a more specific adaptation of postural reactive responses based on the constraints of a voluntary task. Although the elderly used less feedforward adaptation of postural responses based on prior conditions than the young adults, their specific adaptation to immediate change in context assisted their ability to control a difficult manual task. Therefore, although it is thought that postural control deteriorates with aging, healthy elderly people are able to successfully adapt their postural reactive responses to the constraints of the manual task by increasing postural flexibility. One could infer that normal aging does not necessarily deteriorate neural control of postural reactive responses, but changes postural strategies to compensate for other age-related, physiological modifications, in order to adapt to changing environments. The protocol presented here could be used in the future to assess the flexibility of postural reactive responses of fallers to verify whether falls may be related to decreased flexibility to changing constraints of manual tasks.

## Conflict of interest statement

The authors declare that the research was conducted in the absence of any commercial or financial relationships that could be construed as a potential conflict of interest.
